# Comparison of Aspiration Followed by Intra-Lesional Steroid Injection and Surgical Excision in Management of Dorsal Wrist Ganglion

**DOI:** 10.29252/wjps.8.2.181

**Published:** 2019-05

**Authors:** Ajaz Ahmad Shah, Ashiq Hussain Raina, Mudasir Ahmad Ganie, Irshad Ahmad Kumar

**Affiliations:** Department of General Surgery, GMC Srinagar, Jammu and Kashmir, 190010, India

**Keywords:** Aspiration, Intra-lesional, Steroid; Surgery, Excision, Dorsal wrist ganglion

## Abstract

**BACKGROUND:**

About 60-70% of ganglion cysts are found in dorsal part of the wrist. This study compared aspiration followed by intra-lesional steroid (triamcinolone acetate) injection and surgical excision in the management of dorsal wrist ganglion.

**METHODS:**

From Aug 2016 to Aug 2018 in Department of General Surgery, Government Medical College Srinagar, India, 86 Patients with dorsal wrist ganglions were enrolled. The patients were divided to two groups undergoing two different treatment modalities including 68 patients of aspiration with intralesional triamcinolone acetonide injected into the cyst (group A) and 18 patients with surgical excision (group B). Follow up time was 1, 3, 6 and 12 months.

**RESULTS:**

Successful treatment was noticed in 59 patients of group A (86.8%), and in 15 patients of group B (83.3%).

**CONCLUSION:**

Aspiration followed by intra-lesional steroid injection was better managed in comparison to surgical excision.

## INTRODUCTION

Carpal ganglion cyst is the most common benign soft tissue tumor, approximately including 50-70% of the tumors of the hand and wrist area.^1^^-^^3^ They may subside with rest, enlarge with activity, and rupture or disappear spontaneously.^4^ Ganglions are generally seen between the second and fourth decades of life and are more prevalent in women. Patients usually seek medical attention because of the pain, mass, weakness or fear of a malignancy. They are the most common tumor like conditions in the hand and wrist which usually arise from a pedicle in tendon sheath or joint capsule and located over scapholunate ligament.^5^

About 60-70% of ganglion cysts are found in dorsal aspect of the wrist.^5^ There are a number of treatment modalities for ganglion, such as observation, aspiration, intralesional steroid injection, sclerotherapy and surgical excision, but none of these modalities has been the standard or best treatment.^6^^-^^8^ Surgical measures like transfixation, aspiration with seton transfixation, surgical excision and more recently by arthroscopy have been undertaken, but none of them has been the standard or best treatment. As aspiration is still the mainstay of non operative management and most studies demonstrated an approximate success rate of 70%, to improve the results, treatment in aspiration is combined with steroid injection into the ganglion wall. We did this study to compare the effectiveness of the two traditional treatment methods for aspiration followed by intralesional steroid (triamcinolone acetate) injection and surgical excision. 

## MATERIALS AND METHODS

From Aug 2016 to Aug 2018 in Department of General Surgery Government Medical College Srinagar, India, 86 patients with clinical diagnosis of dorsal wrist ganglion were enrolled. The inclusion criteria were dorsal wrist ganglion of at least 1 cm in size, older than 15 years, history of trauma and previous treatment and willingness for follow-up. Diagnosis of ganglion was based on history and clinical examination. In some patients, radiological investigations like X-ray and ultrasonography were done to rule out other lesions. All patients were informed and explained about the lesion and their treatment plan. 

The patients were divided according to their treatment option into two groups. Group A included aspiration followed by intralesional steroid (triamcinolone acetate) injection. Group B that compromised aspiration followed by surgical excision. In group A, under standard aseptic precautions, the ganglion was first infiltrated with 2% xylocaine using 26 G needle and then, aspiration was conducted by 18 G needle and injection of 40 mg triamcinolone acetate applying the same needle port and pre-filled syringe containing the diluted triamcinolone. Crepe bandage was used and the wrist was immobilized for 2 days. 

In group B, the surgical excision was undertaken using similar standard aseptic precautions and after performing local infiltration of 10 mL of 2% xylocaine. In surgical procedure, the entire cyst complex including cyst, pedicle and a cuff of adjacent joint capsule was excised. Follow-up time was 1, 3, 6 and 12 months after treatment. Successful treatment was defined as disappearance of the cyst on final visit of the patient. In case of recurrence, treatment was defined as failure.

## RESULTS

**Fig. 1:**
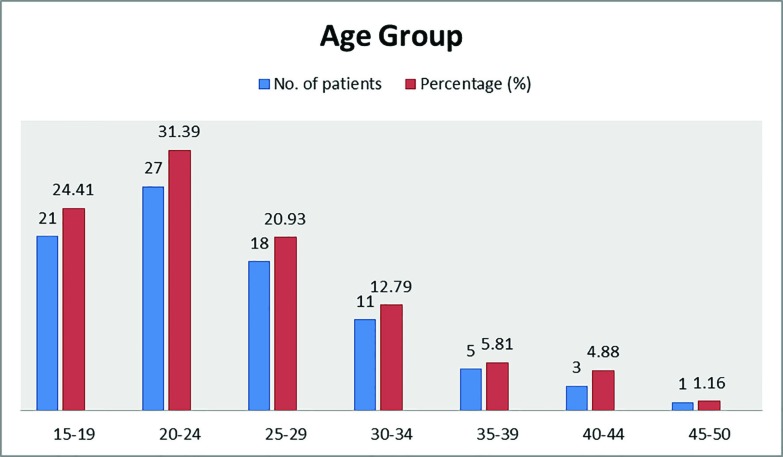
Age distribution of the included patients

**Fig. 2:**
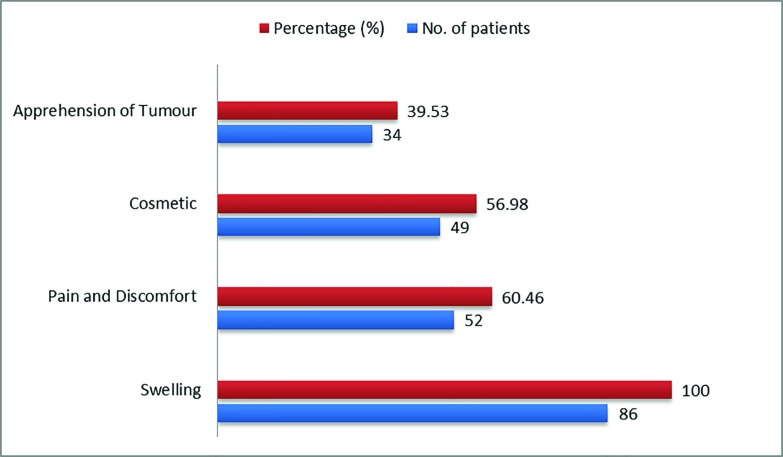
Symptoms of the diseases in included patients

Among the 86 patients, 68 (79.06%) were female and 18 (20.93%) were male patients,while the male/female ratio was 1/3.78. The mean age was 24.8 years (Range: 16-48 years). The age (mean±sd) value of included patients was 24.8±7.69 years with a range of 16-48 years (Figure 1). Swelling was a common presentation in all subjects, while allied complaints synchronous with the swelling were pain and discomfort in 52 (60.46%), cosmetic in 49 (56.98%) and apprehension of tumor in 34 (39.53%) patients (Figure 2). Recurrence was the most common complication of treatment of ganglions. Out of 86 patients, 68 (79.07%) were treated in group A with success rate of 86.76% and recurrence rate of 13.23%. In group B, 18 (20.93%) were treated with success rate of 83.33% and recurrence rate of 16.66%, as shown in Table 1. 

**Table 1:** The success and recurrence rates of all genders regarding both groups of patients

**Group**	**Sex**	**%**	**Success rate** **No. (%)**	**Recurrence rate No. (%)**
A: Aspiration with intralesional triamcinolone acetonide injection	Male: 13 Female: 55 Total: 68	19.12 80.88	59 (86.76%)	9 (13.23%)
B: Surgical excision	Male: 5 Female: 13 Total: 18	27.78 72.22	15 (83.33%)	3 (16.66%)

## DISCUSSION

Carpal ganglion cyst is the most common benign soft tissue tumor, including approximately 50-70% of the tumors of the hand and wrist area.^1^^,^^5^ There are a number of treatment modalities for ganglion such as observation, aspiration, intralesional steroid injection, sclerotherapy and surgical excision, but none of these modalities has been the standard or best treatment. The mean age of our patients at the time of presentation was 24.7 years. Our results are comparable with the findings of Singhal *et al.*^9^ reported a mean age of 25.3 years, and the study by Paul and Sochart demonstrating a mean age of 40.25 years.^10^

Our study involved 86 patients with a male/female ratio of 1/3.78. Similar results were obtained in western regions with a ratio of 1/3.1.^11^ Other studies from the British^12^ and African^13^ population groups reported a ratio of 1/1.4 and 1/1.5, respectively. Recurrence was the most common complication of treatment of ganglions. Paramhans *et al.*^14^ compared two methods of aspiration followed by triamcinolone injection and surgical excision for treatment of wrist ganglions. They found a recurrence rate of 8.4% and 21.5%, respectively and concluded that intracystic steroid injection was a safe mode of treatment.^15^

Humail *et al.* reported a recurrence rate of 43% in aspiration and steroid injection and 24% in surgical excision for treatment of dorsal wrist ganglions.^15^ In a report by Gerhard *et al.* conducted on 38 wrist ganglions, it was found that aspiration has been a better choice than hyaluronidase injection or surgery.^16^ In our study, we found 13.23% recurrence with aspiration followed by steroid injection and 16.66% recurrence was noted after surgical excision. The maximum follow-up time was 1 year.^17^ Janson reported that most of the ganglia recured in first 6 months period.^17^ So aspiration followed by intralesional steroid (triamcinolone acetate) injection was shown to be a better mode of management than surgical excision.

## CONFLICT OF INTEREST

The authors declare no conflict of interest.
